# Tora3D: an autoregressive torsion angle prediction model for molecular 3D conformation generation

**DOI:** 10.1186/s13321-023-00726-8

**Published:** 2023-06-07

**Authors:** Zimei Zhang, Gang Wang, Rui Li, Lin Ni, RunZe Zhang, Kaiyang Cheng, Qun Ren, Xiangtai Kong, Shengkun Ni, Xiaochu Tong, Li Luo, Dingyan Wang, Xiaojie Lu, Mingyue Zheng, Xutong Li

**Affiliations:** 1 Division of Life Science and Medicine, University of Science and Technology of China, Hefei, 230026 Anhui China; 2 Drug Discovery and Design Center, State Key Laboratory of Drug Research, Shanghai Institute of Materia Medica, Chinese Academy of Sciences, 555 Zuchongzhi Road, Shanghai, 201203 China; 3 University of Chinese Academy of Sciences, No.19A Yuquan Road, Beijing, 100049 China; 4 Nanjing University of Chinese Medicine, 138 Xianlin Road, Nanjing, 210023 China; 5 School of Pharmacy, China Pharmaceutical University, 639 Longmian Road, Nanjing, 211198 China; 6 Lingang Laboratory, Shanghai, 200031 China; 7 Precision Pharmacy & Drug Development Center, Department of Pharmacy, Tangdu Hospital, Fourth Military Medical University, Xi’an, 710038 China

**Keywords:** Conformations generation, Autoregressive, Transformer, Deep learning, Small molecules

## Abstract

Three-dimensional (3D) conformations of a small molecule profoundly affect its binding to the target of interest, the resulting biological effects, and its disposition in living organisms, but it is challenging to accurately characterize the conformational ensemble experimentally. Here, we proposed an autoregressive torsion angle prediction model Tora3D for molecular 3D conformer generation. Rather than directly predicting the conformations in an end-to-end way, Tora3D predicts a set of torsion angles of rotatable bonds by an interpretable autoregressive method and reconstructs the 3D conformations from them, which keeps structural validity during reconstruction. Another advancement of our method over other conformational generation methods is the ability to use energy to guide the conformation generation. In addition, we propose a new message-passing mechanism that applies the Transformer to the graph to solve the difficulty of remote message passing. Tora3D shows superior performance to prior computational models in the trade-off between accuracy and efficiency, and ensures conformational validity, accuracy, and diversity in an interpretable way. Overall, Tora3D can be used for the quick generation of diverse molecular conformations and 3D-based molecular representation, contributing to a wide range of downstream drug design tasks.

**Graphical Abstract:**

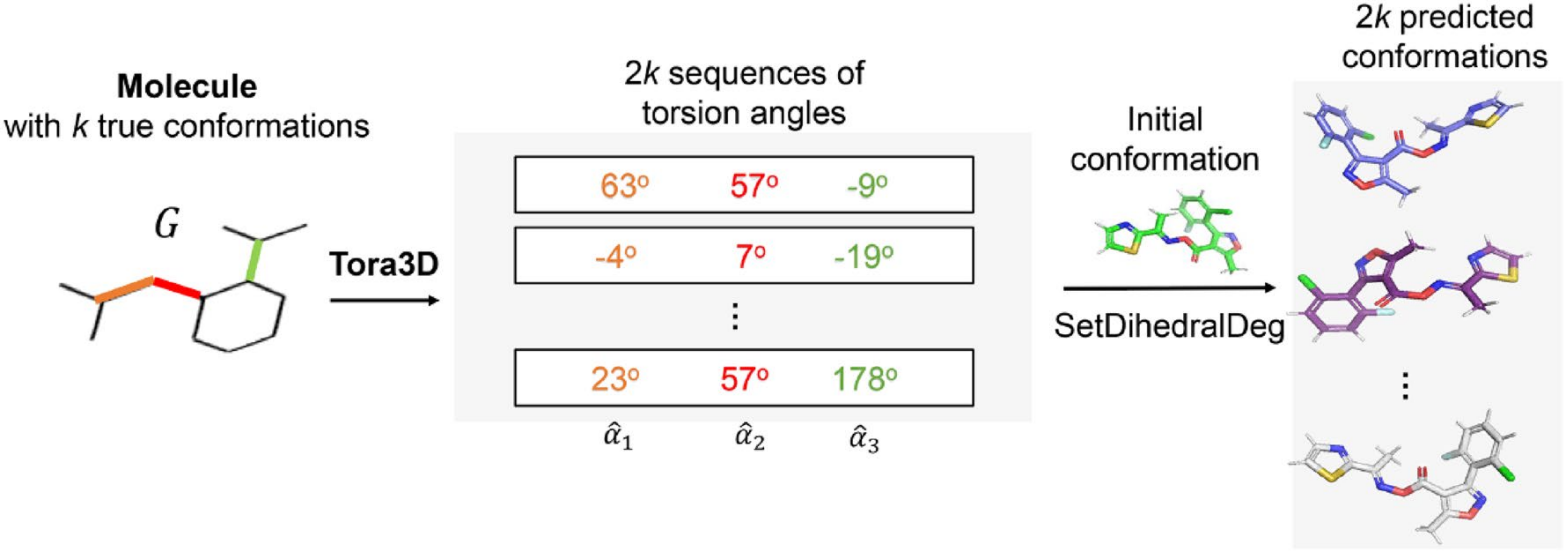

**Supplementary Information:**

The online version contains supplementary material available at 10.1186/s13321-023-00726-8.

## Introduction

Molecular conformation is important for determining a molecule’s chemical and physical properties. Conformation generation is also important in applications such as quantitative structure–activity relationships (QSAR), docking, and virtual screening for drug development [1–5]. The intersection between deep learning and conformation generation has recently drawn attention to their accuracy and efficiency. Deep learning methods generate small molecular conformations set with high accuracy and efficiency, which can accelerate molecular docking and improve its accuracy. Deep learning-based models can also learn molecular representations incorporating 3D structural information, which provides a way forward to improve the predictive modeling of small molecule bioactivities and properties [6].

Over the past decades, generating an accurate 3D structure for a small chemical compound is not trivial. Molecular conformation can be physically determined using X-ray crystallography, but it is prohibitively costly for industry-scale tasks [7]. Ab initio methods can accurately predict molecular geometry, such as density functional theory (DFT) [8], but these approaches usually take up to several hours per small molecule [9]. To handle large-scale molecules, people start turning to classical force fields methods, like UFF [10] or MMFF [11], to estimate conformations, which is efficient but extremely inaccurate [12]. In addition, there are some classical methods to generate low-energy conformations by iteratively enumerating all possible conformations. Systematic search methods such as Monte Carlo simulation (MC) [13], and Distance geometry (DG) [14] are effective in exploring the conformational space, but they can converge to a local minimum rather than the global minimum. Stochastic methods such as Genetic Algorithm (GA) [15] randomly modify the structural parameters of the molecules to increase the probability of finding a global minimum, but the associated computational cost is an important limitation. In systematic search methods, rule-based fast conformational search algorithms such as Omega [3] and Conformator [2] are preferred for sampling large molecular libraries to generate representative conformation ensembles.

Recent deep learning developments hold promise for improving the prediction of the conformation ensembles of small molecules. Generative deep learning can produce structural candidates by predicting possible valid coordinates or distance matrices of a molecule. Since directly generating the 3D coordinates of atoms from the molecular graph like CVGAE [7] faces the problem of SE-(3) invariance, many researchers go for the prediction of the atomic pairwise distances, i.e. distance matrices which are invariant to rotation and translation. GraphDG [16] proposes to model the distribution of inter-atomic distances, while CGCF [17] and ConfVAE [18] take the distribution of distances as intermediate variables to generate conformations. Recently, Ganea et al. [19] further proposed GeoMol to solve the SE-(3) invariance by generating local 3D structures and torsion angles. There are also deep learning models that take an iterative approach to find low-energy conformations. ConfGF [20] directly estimates the gradient field of the log density of the atomic coordinates. GeoDiff [21] uses an SE-(3) equivariant score model to reverse a diffusion process that adds independent Gaussian noise to each atomic coordinate in Euclidean space. These methods can generate a conformation accurately by denoising a point cloud where atoms are in random initial positions but are much more time-consuming. GeoDiff [21] takes about 5000 denoising steps, which costs 9–10 min to generate conformations for a molecule on average.

Although deep learning models have been explored for molecular conformation generation in the hope of combining high accuracy with fast sampling, they typically have the drawback of generating invalid conformations. Most graph neural network (GNN)-based methods fail to learn long-range interactions in graphs, and thus cannot accurately capture dependencies among dihedral angles, which would lead to conflicts among local structures. In addition, it is difficult for distance geometry-based methods to enforce geometric graph constraint [22], hence the accumulated errors in bond angle and length would lead to invalid local structures. To address the problem, systematic search methods assume that bond lengths and some local structures in molecules are essentially constant, and promote slight variations in rotatable bonds to gradually change the conformation of the molecules [23] while avoiding the conflict between the local structures. Recently, some studies have also proposed that rotatable bonds play a crucial role in determining the conformation of molecules, such as Torsion Library [24] and TorsionNET [25].

Here, we build a deep learning model, namely Tora3D, to predict the torsion angles combinations of all rotatable single bonds in a molecule from a 2D molecular graph, to obtain the set of predicted conformations. Like systematic approaches, our methodology follows a basic assumption that the conformational space mainly originates from the rotation of single bonds in the molecule, while keeping bond lengths and angles [23] constant. We replace the time-consuming and compute-intensive iterative process of rotatable bonds in a systematic method with an autoregressive deep learning model. The combination of deep learning and prior knowledge guarantees the accuracy, speed and validity of conformation generation while avoiding the disadvantages of the systematic method. The framework of Tora3D is designed to address the problems inherent in previous methods: (1) An autoregression neural network with an attention mechanism can guarantee the overall structural validity of the molecular conformation. The autoregressive neural network predicts the torsion angles of rotatable bonds in a molecule one by one. Hence, Tora3D could consider the dependencies among each dihedral angle to avoid clashes among local structures, and the attention mechanism can explain the dependencies and ensure spatial rationality. (2) Reconstructing the conformation by a two-stage generation procedure can guarantee the local structural validity in molecular conformation. Tora3D utilizes predicted torsion angles to assemble valid local structures that were constructed of bond lengths and angles determined by standard cheminformatics tools. Compared with directly generating conformations in an end-to-end way, the two-stage generation procedure of Tora3D can significantly reduce the dimensionality of the sample space and avoid local structural invalidity caused by wrong bond lengths and angles. (3) Tora3D could generate a set of relatively low-energy molecular conformations quickly by giving relative energies when making inferences. Overall, Tora3D aims at achieving a balance among three aspects of performance in the conformational generation including accuracy, validity, and diversity.

## Method

### Notation

Firstly, the symbols and notations used here were summarized in Table 1. $G=\left(V,E\right)$ represents a molecular graph, in which $V=\{{v}_{1},{v}_{2},\dots ,{v}_{|V|}\}$ is the set of feature vectors of atoms (nodes) and $E=\left\{{e}_{ij}\right|(i,j)\in V\times V\}$ is the set of feature vectors of bonds (edges). The atomic and bond feature vectors were drawn from the input features employed by AttentiveFP, a molecular structural representation scheme based on the graph attention mechanism [26]. The ${h}_{v}^{0}$ and ${h}_{v}^{T}$ represent the initial and updated atomic representations, respectively. The ${\alpha }_{l}$ represents a true normalized torsion angle value (the normalized operation will be discussed later) and ${\widehat{\alpha }}_{l}$ is a predicted one. The $A=\{{\alpha }_{1}, {\alpha }_{2}, {\alpha }_{3}, {\alpha }_{4}\dots ,{\alpha }_{l}, \dots \}$ and $\widehat{A}=\{\widehat{{\alpha }_{1}}, \widehat{{\alpha }_{2}}, \widehat{{\alpha }_{3}}, \widehat{{\alpha }_{4}},\dots , \widehat{{\alpha }_{l}}, \dots \}$ represents the sequence of true and predicted normalized torsion angle values, respectively. Both ${\tau }_{l}^{0}$ and ${\tau }_{l}$ represent the torsion angle representations, where ${\tau }_{l}^{0}$ represents the torsion angle obtained by the Torsion representation module and used as the initial input of the Transformer module, while ${\tau }_{l}$ denotes the updated torsion angle representation (these two modules will be described below). The $T^{0} = \left\{ {\tau_{1}^{0} , \tau_{2}^{0} , \tau_{3}^{0} , \tau_{4}^{0} , \ldots ,\tau_{l}^{0} , \ldots } \right\}$ and $T = \,\left\{ {\tau_{1} , \tau_{2} , \tau_{3} , \tau_{4} , \ldots ,\tau_{l} , \ldots } \right\}$ denotes all torsion angle representations of a molecule, namely the sequence of torsion angle representations. The *k* denotes the true number of conformations of a molecule.

**Table 1:** List of symbols and notations used in the paper

Symbol	Description
$G$	The molecular graph
$V=\{{v}_{1},{v}_{2},\dots ,{v}_{|V|}\}$	The set of feature vectors of atoms (nodes)
$E=\left\{{e}_{ij} \right| (i,j)\in V\times V\}$	The set of feature vectors of bonds (edges)
${h}_{v}^{t}$	The representation of the atom $v$ in the $t$ layer
${\alpha }_{l}$	A true normalized torsion angle value
${\widehat{\alpha }}_{l}$	A predicted normalized torsion angle value
$A=\{{\alpha }_{1 }, {\alpha }_{2 }, {\alpha }_{3 }, {\alpha }_{4 ,}\dots ,{\alpha }_{l }, \dots \}$	The sequence of true normalized torsion angle values
$\widehat{A}=\{\widehat{{\alpha }_{1}}, \widehat{{\alpha }_{2}}, \widehat{{\alpha }_{3}}, \widehat{{\alpha }_{4}},\dots , \widehat{{\alpha }_{l}},\dots \}$	The sequence of predicted normalized torsion angle values
${\tau }_{l}^{0}$ or ${\tau }_{l}$	The torsion angle representation
$T^{0} = \left\{ {\tau_{1}^{0} , \tau_{2}^{0} , \tau_{3}^{0} , \tau_{4}^{0} , \ldots ,\tau_{l}^{0} , \ldots } \right\}\,{\text{or}}\,T = \left\{ {\tau_{1} , \tau_{2} , \tau_{3} , \tau_{4} , \ldots ,\tau_{l} , \ldots } \right\}$	The sequence of torsion angle representations
*k*	The true number of conformations of a molecule

### Overview

**Fig. 1:**
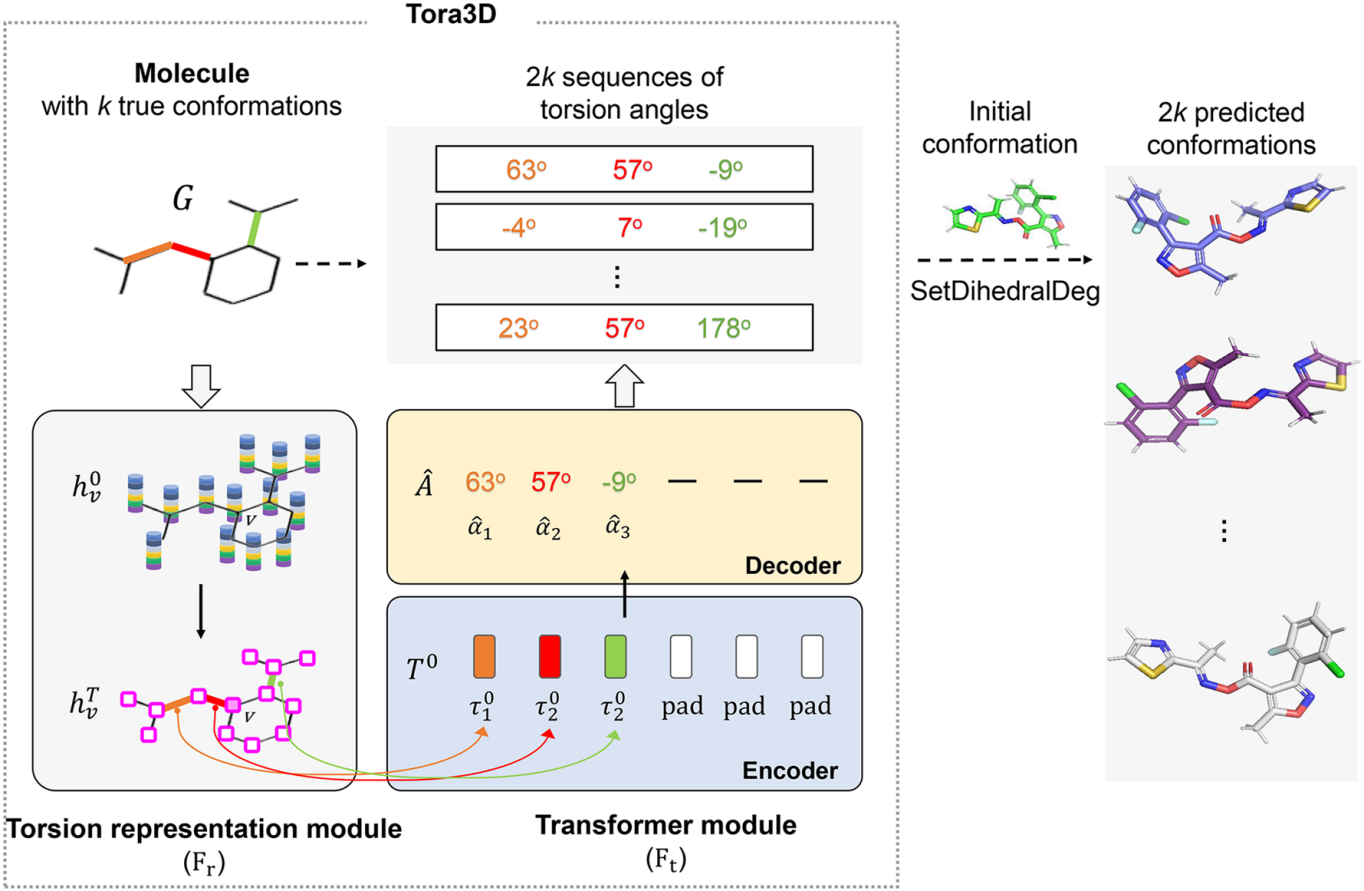
The framework of Tora3D and the usage of it to generate small molecule conformations. For the case molecule with 3 rotatable bonds (orange, red and green), Tora3D generates 2* k* sequences of 3 torsion angles, which can be used to rebuild 2* k* predicted conformations

In this section, we want to outline our approach. Tora3D is a neural network model that can predict a series of sequences of torsion angles of all rotatable single bonds in a molecule from a 2D molecular graph (Fig. 1). Inputting a molecular graph (containing information about nodes and edges, as well as topology), Tora3D was trained to predict all torsion angle values of the molecule. Tora3D is divided into two parts: Torsion representation module (${\mathrm{F}}_{\mathrm{r}}$) and Transformer module (${\mathrm{F}}_{\mathrm{t}}$). The former obtains the sequence of torsion angle representations (T^0^) of the molecule from the 2D molecular graph (G) (Eq. 1), and the latter obtains the sequence of normalized torsion angle values $\widehat{A}$ from (T^0^) (Eq. 2). Once the torsion angle values have been predicted by Tora3D, they can be used to rebuild conformations of the small molecule from the initial conformation (Fig. 1).

1 $$T^{0} = {\text{F}}_{{\text{r}}} \left( G \right)$$

2 $$\widehat{{\text{A}}}\,{ = }\,{\text{F}}_{{\text{t}}} \left( {T^{0} } \right)$$

**Fig. 2:**
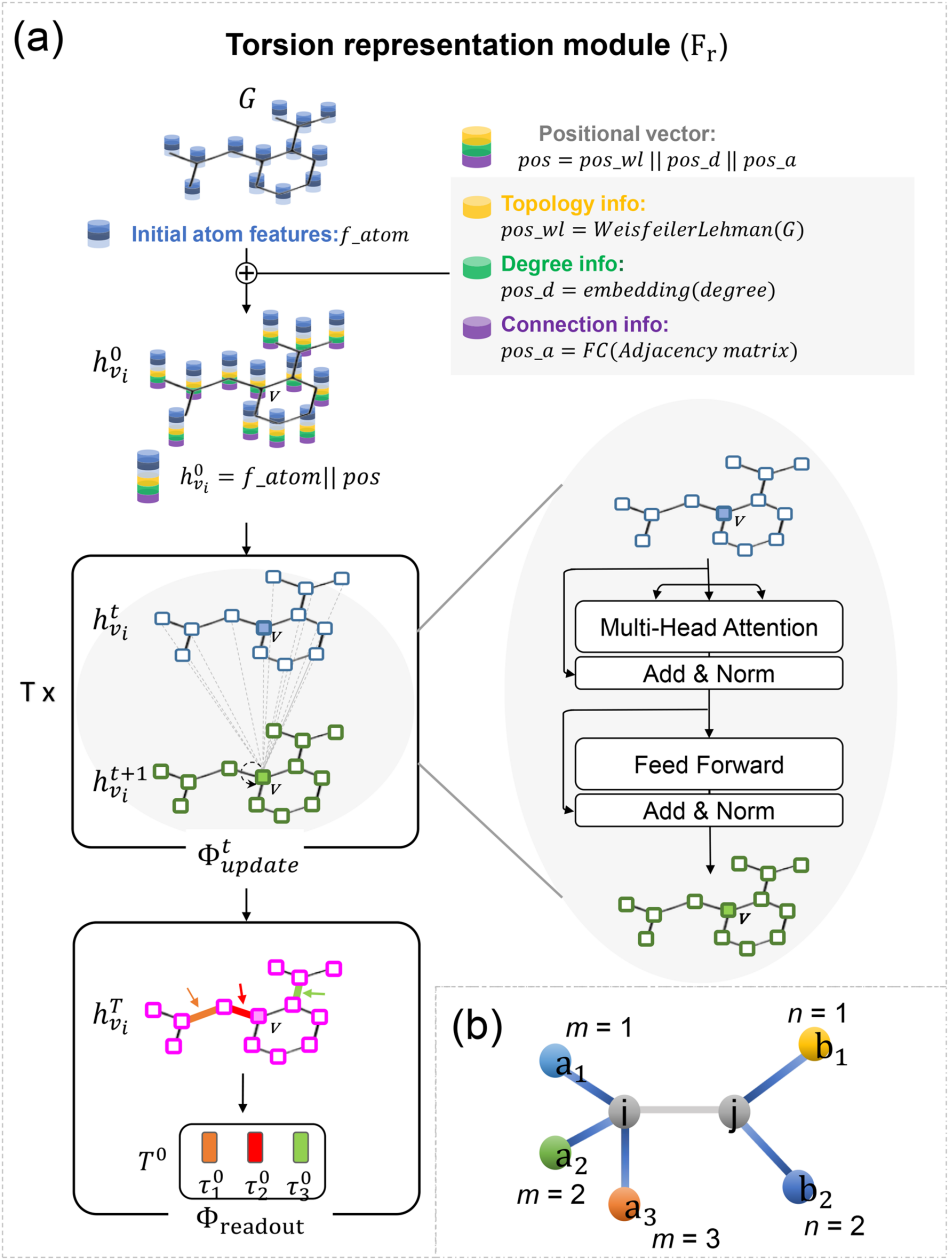
**a** Torsion representation module (${\mathrm{F}}_{\mathrm{r}}$). **b** The calculation of the normalized torsion angle $\alpha$ of bond ij. There are three options for end atom a and two options for end atom b in this case

To avoid over parametrization, the normalized torsion angle defined by Ganea et al. was used here, which is uniquely determined independent of the choice of terminal atoms [19]. Specifically, the normalized torsion angle ${\alpha }_{l}$ is calculated as Eq. 3 to Eq. 5.

3 $$s_{{{\text{a}}_{m} {\text{b}}_{n} }} \underline{\underline{{{\text{def}}}}} \left[ {\frac{{\cos \left( {\Delta_{{{\text{a}}_{m} {\text{b}}_{n} }} } \right)}}{{\sin \left( {\Delta_{{{\text{a}}_{m} {\text{b}}_{n} }} } \right)}}} \right]$$

4 $$s\,\underline{\underline{{{\text{def}}}}} \,\sum {_{m,n} c\, \cdot s_{{a_{m} b_{n} }} }$$

5 $$\alpha_{l} \,\underline{\underline{{{\text{def}}}}} - arctan\,\left( {\frac{s}{\left\| s \right\|}} \right)$$

where ${}_{{\mathrm{a}}_{m}{\mathrm{b}}_{n}}$ refers to the angle of twist with terminal atom ${\mathrm{a}}_{m}$ and ${\mathrm{b}}_{n}$ as shown in Fig. 2b. And $c$ is a constant, to avoid ${s}_{{\mathrm{a}}_{m}{\mathrm{b}}_{n}}$ canceling each other out due to summation. It has been demonstrated that when a rotatable bond rotates by an angle γ, the normalized torsion angle α correspondingly rotates γ [19].

### Torsion representation module

The first part of Tora3D is the Torsion representation module (${\mathrm{F}}_{\mathrm{r}}$**)** (Fig. 2a), which obtains the sequence of torsion angle representations (T^0^) of the molecule from the 2D molecular graph (***G***) (Eq. 1). First, the position information $pos$ is concatenated to the initial feature vectors $v$ of each atom to obtain the initial representation ${h}_{v}^{0}$ of each atom (Eq. 6 and Eq. 7). Then, the initial representations {${h}_{{v}_{1}}^{0}, {h}_{{v}_{2}}^{0},\dots ,{h}_{{v}_{|V|}}^{0}$} of the atoms are put into self-attention based ${\Phi }_{\mathrm{update}}$ to updates the atomic representations denoted as ${h}_{v}^{T}$ (Eq. 8). Finally, the updated atomic representations are combined with corresponding edges' representations to obtain the initial representation ${\tau }_{l}^{0}$ for each torsion angle by ${\Phi }_{readout}$ (Eq. 9 to Eq. 12). Following are the detailed algorithms.

The position vector ($pos$) in Eq. 7 consists of three parts to ensure that it can contain the position information of nodes throughout the molecular graph. The first part $pos\_wl=WeisfeilerLehman(G)$ is a vector calculated using the Weisfeiler-Lehman (WL) algorithm [27], which is used to detect graph isomorphism. By WL, nodes with the same topology have the same $pos\_wl$. The second part $pos\_d=embedding(degree)$ is the degree’s embedding of the atom. The third part $pos\_a=FC(Adjacency matrix)$ is the position vector representation of each atom obtained from the adjacency matrix that can provide connection information of the graph.

Concatenating these three parts (Eq. 6), the final position vector $pos$ replaces the position scalar in the original transformer added to each token, and it is concatenated with the initial feature vectors $v$ of an atom to obtain the initial representation ${h}_{v}^{0}$ of each atom (Eq. 7).

6 $$pos = pos\_wl\left\| {pos\_d} \right.\left\| {pos\_a} \right.$$

7 $$h_{v}^{0} = v\left\| {pos} \right.$$

${\Phi }_{update}^{t}$(Eq. 8) is the atomic update module that updates the initial representation of each atom, i.e. {${h}_{{v}_{1}}^{0},{h}_{{v}_{2}}^{0},\dots ,{h}_{{v}_{|V|}}^{0}$}. The algorithm is borrowed from the self-attention module in the transformer to overcome the difficulties of traditional GNN’s long-distance messaging. After T (hyperparameter, Additional file 1: Table S1) times, the updated representations {${h}_{{v}_{1}}^{T},{h}_{{v}_{2}}^{T},\dots ,{h}_{{v}_{|V|}}^{T}$} were obtained, in which each atom interacts with all other atoms of the graph through attention.

8 $$h_{{v_{1} }}^{t + 1} ,h_{{v_{2} }}^{t + 1} , \ldots ,h_{{v_{\left| V \right|} }}^{t + 1} = {\Phi }_{update}^{t} \left( {h_{{v_{1} }}^{t} ,h_{{v_{2} }}^{t} , \ldots ,h_{{v_{\left| V \right|} }}^{t} } \right)\, = self - attention\left( {h_{{v_{1} }}^{t} ,h_{{v_{2} }}^{t} , \ldots ,h_{{v_{\left| V \right|} }}^{t} } \right)$$

Given the updated atomic representations $H=\{{h}_{{v}_{1}}^{T},{h}_{{v}_{2}}^{T},\dots ,{h}_{{v}_{|V|}}^{T}\}$ and the edge information $E$, the initial representations of torsion angles $T^{0} = \left\{ {\tau_{1}^{0} , \tau_{2}^{0} , \tau_{3}^{0} , \tau_{4}^{0} , \ldots ,\tau_{l}^{0} , \ldots } \right\}$ are obtained by ${\Phi }_{\mathrm{readout}}$ (Eq. 9).

9 $$T^{0} = {\Phi }_{readout} \left( {H,E} \right)$$

Specifically, the representation of each torsion angle ${\tau }_{l}^{0}$ is obtained by integrating information about the neighboring edges of each rotatable bond and the corresponding atoms (Eq. 10).

10 $$\tau_{1}^{0} = h_{atoms} \left\| {e_{edges} } \right.$$

Here, ${e}_{edges}$ for each edge is the concatenated information of itself ${e}_{ij}$ with its neighboring edges ${e}_{ai}$ and ${e}_{bj}$ (Eq. 11).

11 $$e_{edges} = e_{ai} \left\| {e_{ij} } \right.\left\| {e_{bj} } \right.$$

For example, as shown in Fig. 2b, $$e_{ai} = {\raise0.7ex\hbox{${\left( {e_{{a_{1} i}} + e_{{a_{2} i}} + \ldots } \right)}$} \!\mathord{\left/ {\vphantom {{\left( {e_{{a_{1} i}} + e_{{a_{2} i}} + \ldots } \right)} {\left| {e_{ai} } \right|}}}\right.\kern-0pt} \!\lower0.7ex\hbox{${\left| {e_{ai} } \right|}$}}$$ is the integrated representation of edges between atom i and atom $${\mathrm{a}}_{1}$$, $${\mathrm{a}}_{2}$$ and $${\mathrm{a}}_{3}$$, and $$e_{bj} = \,{\raise0.7ex\hbox{${\left( {e_{{b_{1} j}} + e_{{b_{2} j}} + \ldots } \right)}$} \!\mathord{\left/ {\vphantom {{\left( {e_{{b_{1} j}} + e_{{b_{2} j}} + \ldots } \right)} {\left| {e_{bj} } \right|}}}\right.\kern-0pt} \!\lower0.7ex\hbox{${\left| {e_{bj} } \right|}$}}$$ denotes the integrated representation of edges between atom j and $${b}_{1}$$ and $${b}_{2}$$.

Similarly, the two atoms of a rotatable bond concatenate information about themselves $${h}_{i}$$ and $${h}_{j}$$, with all terminal atoms $$h_{a\,} \, = \,{\raise0.7ex\hbox{${\left( {h_{{a_{1} }} + h_{{a_{2} }} + \ldots } \right)}$} \!\mathord{\left/ {\vphantom {{\left( {h_{{a_{1} }} + h_{{a_{2} }} + \ldots } \right)} {\left| {h_{a} } \right|}}}\right.\kern-0pt} \!\lower0.7ex\hbox{${\left| {h_{a} } \right|}$}}$$ and $$h_{b} \, = \,{\raise0.7ex\hbox{${\left( {h_{{b_{1} }} + h_{{b_{2} }} + \ldots } \right)}$} \!\mathord{\left/ {\vphantom {{\left( {h_{{b_{1} }} + h_{{b_{2} }} + \ldots } \right)} {\left| {h_{a} } \right|}}}\right.\kern-0pt} \!\lower0.7ex\hbox{${\left| {h_{a} } \right|}$}}$$ to obtain the representation of atoms $${h}_{atoms}$$ (Eq. 12).

12 $$h_{{{\text{atoms}}}} { = }h_{{\text{a}}} \left\| {h_{{\text{i}}} } \right.\left\| {h_{j} } \right.\left\| {h_{{\text{b}}} } \right.$$

Thus, by the above process of ${\Phi }_{readout}$, the initial representation of torsion angles T^0^ is obtained from the overall atomic representation of each side of a rotatable bond. Unlike some models that predict atomic extrinsic coordinates of a molecule from atom representation, Tora3D is SE (3)-invariant by focusing on torsional space specific to the molecule (intrinsic coordinates).

### Transformer module

**Fig. 3:**
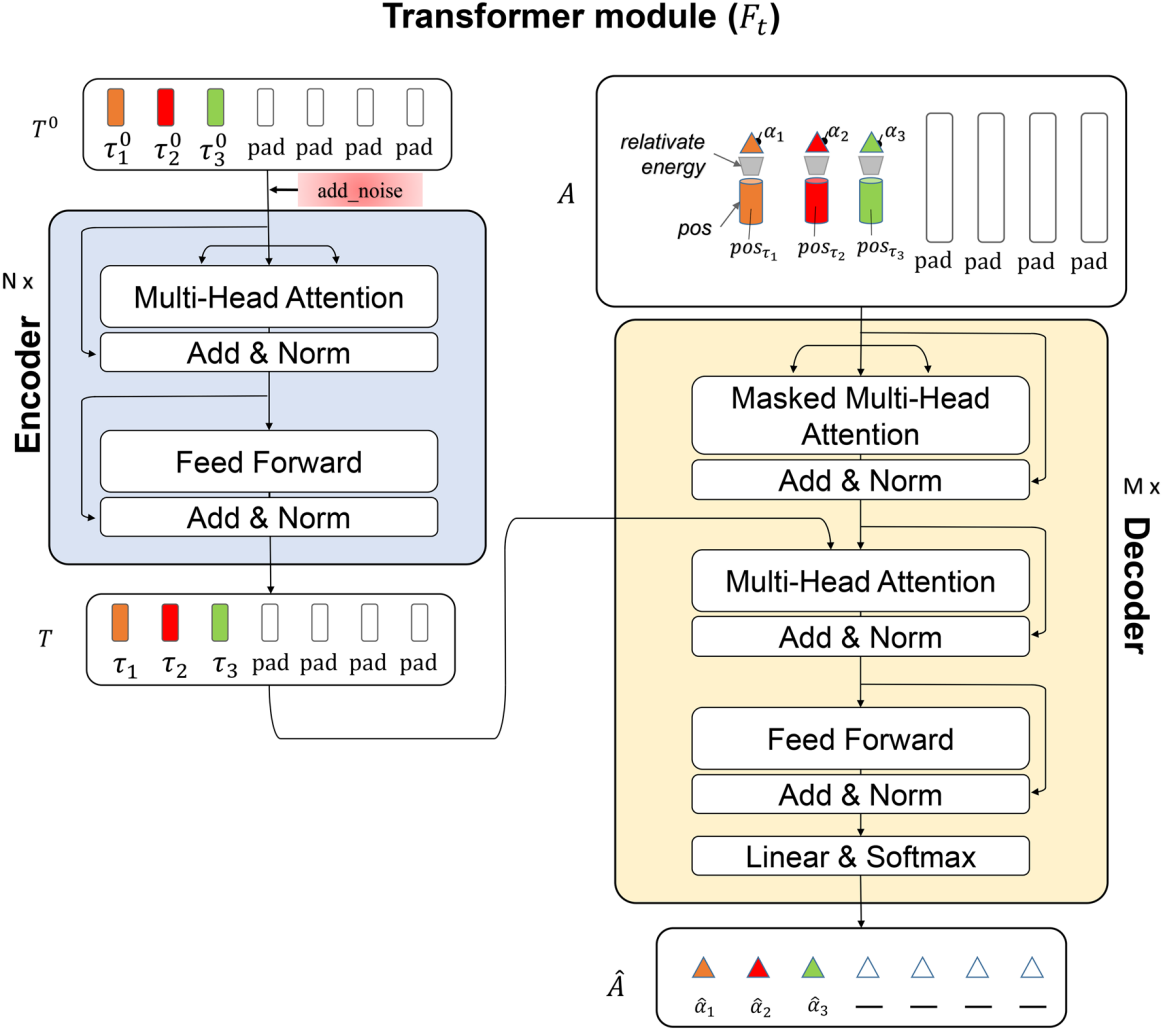
The transformer module has an encoder-decoder structure that uses stacked self-attention and fully connected layers. The sequence of initial torsion angle representations T^0^ was input into the encoders (left) and updated for N (hyperparameter, Additional file 1: Table S1) times to obtain a sequence of continuous representations T. Given T, the M (hyperparameter, Additional file 1: Table S1) stack of decoders (right) generates an output sequence, i.e., normalized torsion angle values $\widehat{A}$, one element at a time. At each step the model is autoregressive, consuming the previously generated angle values as additional input when generating the next

The Transformer module (${\mathrm{F}}_{\mathrm{t}}$) (Fig. 3) is used to accept the sequence of torsion angle representations T^0^ as input and output the sequence of predicted torsion angle values $\widehat{A}$ (Eq. 2). Compared to the original Transformer’s framework, the Transformer module has a few changes as detailed below.

#### Transformer encoder

Transformer encoder maps an input sequence of torsion angle representations T^0^ to the sequence of updated torsion angle representations T (Fig. 3). Gaussian noise (hyperparameter, Additional file 1: Table S1) with a mean of zero and standard deviation of 5.0, which is determined by hyperparameter searching, was added to T^0^ to allow the model generates multiple conformations by introducing an element of randomness. The position coding of the original Transformer was removed since ${\tau }_{l}^{0}$ already contains the position information, as defined in the Torsion representation module.

#### Transformer decoder

Given T, the decoder generates the sequence of predicted normalized torsion angle values $\widehat{A}$ (Fig. 3). The model predicts each $\widehat{A}$ with reference to the previously predicted torsion angle values i.e., $\widehat{a}$, and their corresponding position encoding (${pos}_{{\tau }_{i}}$), as well as the relative energy (kcal/mol) for each molecular conformation. Such an autoregressive approach avoids local structural clashes. Moreover, the relative energy as input also allows the model to generate energy-specific conformations.

### Conformation generation

As shown in Fig. 1, Tora3D predicts the torsion angle value of rotatable bonds consisting of heavy atoms. Once the torsion angles of all the rotatable bonds of a small molecule have been predicted, the fragments of the small molecule can be assembled to form the overall conformation. There are already some accurate and effective knowledge-based algorithms for generating conformation ensembles from fragments have been demonstrated, represented by the commercial algorithm Omega [3] and the freely available algorithm Conformator [2]. Thus, we directly use one of the conformations generated by the Conformator as the initial conformation and twist it to obtain the predicted conformation based on the torsion angle values predicted by the Tora3D. The comparison between the initial conformations and Tora3D’s generated conformations are shown in supporting information, Additional file 1: Figure S4 and Additional file 1: Table S4. To be specific, we reset the torsion angles of an initial conformation by the SetDihedralDeg function in RDKit [28].

### Experiments

#### Dataset and split

Following previous works [17, 20], the Geometric Ensemble Of Molecules (GEOM)-Drugs dataset was used for building the model. The GEOM-drugs dataset contains 118,434,901 molecular conformations of 304,466 unique molecules, generated by advanced sampling and semi-empirical DFT. Relative energy of each conformation is also included in GEOM-drugs, which is the difference between the absolute energy of a conformation and that of the lowest-energy conformation. A value of 0 kcal/mol signifies the energy of the lowest-energy conformation. The molecules in GEOM-Drugs are annotated by experimental data related to biophysics, physiology, and physical chemistry [1]. The test set of Shi et al. containing 200 molecules was also used here for performance evaluation (test set I) [20]. Analysis showed that the number of conformations of each molecule in test set I is less than 100, while the number of conformations of each molecule in the GEOM-Drugs dataset ranges from 0 to 12,000 (Additional file 1: Figure S1). Thus, Test set I is not reflective of the overall modeling dataset. Therefore, an additional test set II was collected, which contains randomly selected 1000 molecules and their conformation number ranges from 0 to 500, same as the range of conformation number of molecules in the GEOM-Drugs dataset. With a similar distribution to the entire dataset, test set II consists of more conformations with higher diversity than test set I, which was used to further evaluate model performance affected by conformational flexibility.

#### Evaluation indicators

Coverage (COV) and Matching (MAT) score are used to measure the diversity and accuracy respectively. COV score reports the percentage of reference conformers that are produced by the predicted ensemble. MAT score reports the minimum RMSD between a generated conformer and the references. Following the conventional Recall measurement, COV-R and MAT-R can be defined as [21]:

13 $${\text{COV - R}}\left( {S_{g} ,{ }S_{r} } \right) = \frac{1}{{\left| {S_{r} } \right|}}\left| {\left\{ {R \in S_{r} {|}RMSD\left( {R,\hat{R}} \right) < \delta , R \in S_{g} } \right\}} \right|$$

14 $${\text{MAT - R}}\left( {S_{g} ,{ }S_{r} } \right) = \frac{1}{{\left| {S_{r} } \right|}}\mathop \sum \limits_{{R \in S_{r} }} \mathop {\min }\limits_{{\hat{R} \in S_{g} }} RMSD\left( {R,\hat{R}} \right)$$

Here, ${S}_{g}$ is the set of generated conformations and ${S}_{r}$ is the set of reference conformations of a molecule. $\widehat{R}$ and $R$ refer to a generated conformation and a reference conformation, respectively. The $\delta$ is set as 1.25. The above equations are used for calculating COV-R and $\mathrm{MAT}$-R (Recall). And to calculate COV-P and $\mathrm{MAT}$-P (Precision), ${S}_{g}$ and ${S}_{r}$ should be swapped. Generally, higher COV rates or lower MAT score suggest that more realistic conformations are generated. And the Recall metrics concentrate more on the diversity, while the Precision metrics depend more on the quality. Consistent with previous work, we predicted and generated twice as many conformations as the number of true conformations for each molecule.

## Results

### Model performance in conformational diversity and accuracy

We have compared Tora3D with several recent popular models of molecular 3D conformation prediction: CVGAE [7], GraphDG [16], CGCF [17], ConfVAE[18], GeoMol [19], ConfGF [20], and GeoDiff [21]. In addition, we have conducted the comparisons with torsion angle prediction methods including Torsion Library [24] and TorsionNET [25]. The implementation is shown in supporting information, Additional file 1: Figure S3 and Additional file 1: Table S3. As shown in Table 2, Tora3D shows superior performance compared to the above-mentioned models in conformational diversity (higher COV) and accuracy (lower MAT) on the same test set (Test set I) that was reported in Shi et al. [20]. Although its COV-R is slightly lower than GeoDiff, Tora3D makes a trade-off between accuracy and efficiency. Tora3D is relatively fast and can predict all conformations of a molecule within 5 to 8.4 s, while GeoDiff needs about 10 min for a molecule.

**Table 2:** Performance comparison of models on the GEOM-drugs dataset (Test set I)

Models	COV-R(↑)	MAT-R(↓)	COV-P(↑)	MAT-P(↓)	Speed (s/molecule)
CVGAE	0.00	3.0702	–	–	–
GraphDG	8.27	1.9722	2.08	2.4340	–
CGCF	53.96	1.2487	21.68	1.8571	-
ConfVAE	55.20	1.2380	22.96	1.8287	10–16
GeoMol	67.16	1.0875	–	–	1–4
ConfGF	62.15	1.1629	23.42	1.7219	> 600
GeoDiff	**82.96**	0.9525	48.27	1.3205	540–600
Tora3D	80.37	**0.9272**	**62.22**	**1.1524**	**5–8.4**

^*^The results of CVGAE[7], GraphDG[16], CGCF[17], ConfGF[20], and GeoDiff[21] are borrowed from Shi et al.[20]. The experiments of ConfVAE and GeoMol[19] were implemented by ourselves. The inference speed for a molecule of each model was tested by ourselves

Position embedding is devised to capture the position/location of the node within the broader context of the graph structure to tackle the problem that conventional GNN architecture hardly learns long-range patterns in graphs. The importance of the position embedding in Tor3D is verified by an ablation experiment by removing the position embedding from the model or replacing it with a learnable position embedding that represents the position of atoms. It can be seen in Table 3 that Tora3D with the specially designed position embedding provides better performance, especially on conformation accuracy and coverage. The results in Table 3 demonstrate that removing positions embedding for nodes in Tora3D, which is just like a conventional GNN architecture, does harm the quality of conformation generation. And our strategy addresses the issue of capturing long-range node dependencies, leading to better accurate and diverse conformations than learnable-position embedding.

**Table 3:** Performance of different position embedding

Models	COV-R(↑)	MAT-R(↓)	COV-P(↑)	MAT-P(↓)
Without position embedding	57.32	1.1742	62.05	1.4200
Learnable-position embedding	73.21	1.0109	**62.85**	1.4459
Tora3D	**81.92**	**0.9297**	62.16	**1.1600**

**Fig. 4:**
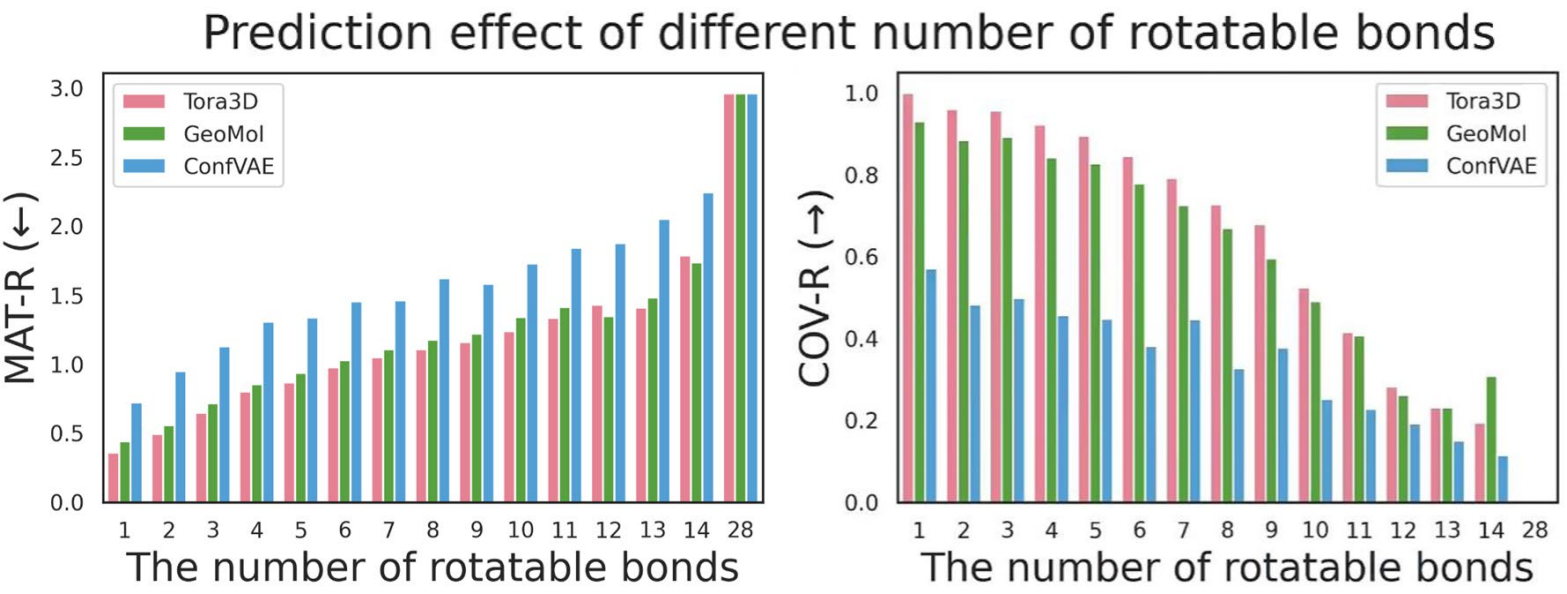
Prediction performance of the model for different numbers of rotatable bonds. The x-axis indicates the number of rotatable bonds, and the y-axis indicates the prediction performance

In addition, Tora3D uses a basic assumption same to systematic methods that the only factor changing the conformation of a molecule is rotatable bonds. The number of rotatable bonds (nRotb) plays a decisive role in molecular flexibility, as the space of possible conformations grows exponentially with it. Thus, nRotb would affect the prediction performance (Fig. 4). We have implemented ConfVAE and GeoMol [19] to test the effect of nRotb based on test set II. Here ConfVAE and GeoMol were selected for comparison because they showed relatively good performance and acceptable speed (Table 2). As shown in Fig. 4, for each of the models, essentially, the more rotatable bonds a molecule has, the more difficult it is to predict its conformations. More importantly, the performance of Tora3D consistently surpasses ConfVAE and GeoMol when the molecule has less than 10 rotatable bonds (Table 4), which is also an important criterion for the drug-likeness of a molecule [29].

**Table 4:** Performance comparison of models on the GEOM-drugs dataset (Test set II)

Models	COV-R(↑)	MAT-R(↓)	COV-P(↑)	MAT-P(↓)
ConfVAE	40.06	1.3771	-	-
GeoMol	72.50	1.1000	61.15	1.2009
Tora3D	**81.92**	**0.9297**	**62.16**	**1.1600**
ConfVAE (nRotb ≤ 10)	42.43	1.3296	–	–
GeoMol (nRotb ≤ 10)	76.36	0.9380	57.29	1.1611
Tora3D (nRotb ≤ 10)	**83.03**	**0.8704**	**63.81**	**1.0906**

### Conformational validity

Given that some inherent defects in typical conformation prediction models would cause conformational invalidity and undermine model performance, we have introduced some strategies into Tora3D to improve its validity.

**Fig. 5:**
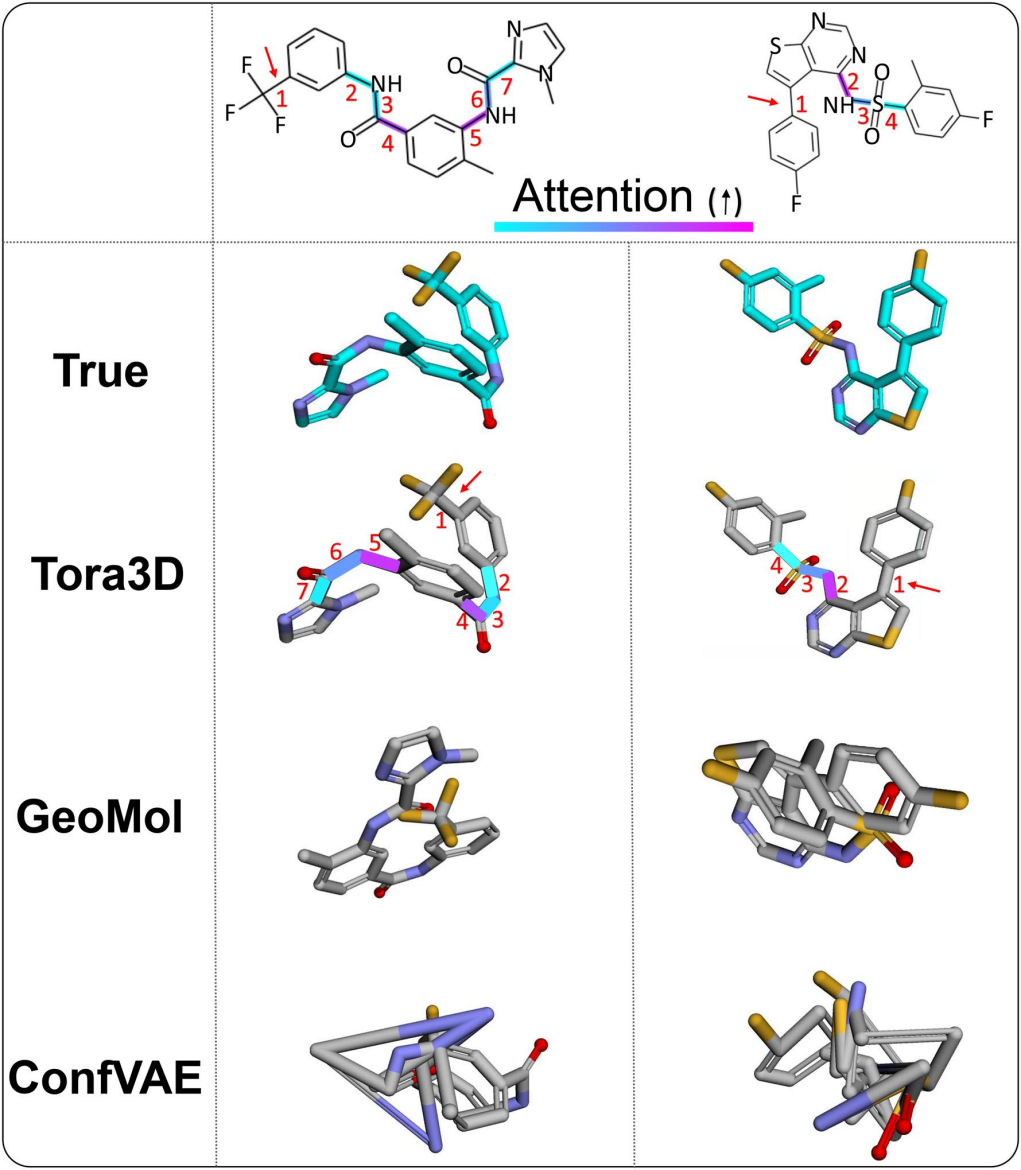
Comparisons of the conformations predicted by Tora3D, GeoMol, and ConfVAE. The 2D molecule graphs in the first row are marked by the score of attention paid by one of the torsion angles to other bonds (i.e., the bond pointed by the red arrow). Their attention scores toward other torsion angles are indicated by the highlighting. Bonds colored magenta refer to high attention and cyan refer to low attention

The first and most important strategy of Tora3D is its autoregressive algorithm. Tora3D predicts the torsion angles of rotatable bonds in a molecule one by one to avoid arising conflicts among local structures, and thus the current torsional angle value is determined not only by the molecular graph but also by the previously predicted torsion angle values. Most deep learning-based conformation generation models do not consider the dependencies among the local structures of a molecule and predict each dihedral angle as an independent variable, which will inevitably lead to invalidity of the overall molecular conformation. For example, topologically distant fragments of a molecule may conflict with each other in space. Figure 5 shows examples of the torsion angle predictions of Tora3D, GeoMol and ConfVAE. With the autoregressive algorithm, Tora3D could consider every rotatable bond sequentially to avoid clashes among local structures, but GeoMol and ConfVAE can not explicitly capture the global interactions as the torsion angles are predicted independently.

At the same time, the autoregressive approach could further ensure the spatial rationality of the whole molecule by attention mechanism. As shown in Fig. 5, the 1th bond of the first molecule shows higher attention with respect to the more distant 5th and 4th bonds but lower attention to the closer 2nd bond. The attention scores are consistent with the observation that the incorrect rotations of the 5th and 4th bond would cause the spatial conflict between the trifluoromethyl and 1-methylimidazole, and thus the 1st bond have a stronger relation to the 5th and 4th bond torsion angle than the closer 2nd bonds. In the second molecule, the 1st bond shows higher attention to the 2nd bond, whose improper rotation would cause serious spatial conflict between the terminal structures in the molecule.

The other strategy is to incorporate prior knowledge of local 3D structures of each non-terminal atom, to ensure the validity of conformational generation. The main challenge in molecular conformation generation comes from the enormous size of the 3D structure space consisting of bond lengths, bond angles, and torsion angles. However, the molecular graph imposes specific constraints on the set of possible stable local structures, which can be predicted by fast cheminformatics methods. Thus, Tora3D incorporates the prior knowledge about bond lengths and angles to guarantee validity by assembling fixed local structures directly from an initial conformation.

**Fig. 6:**
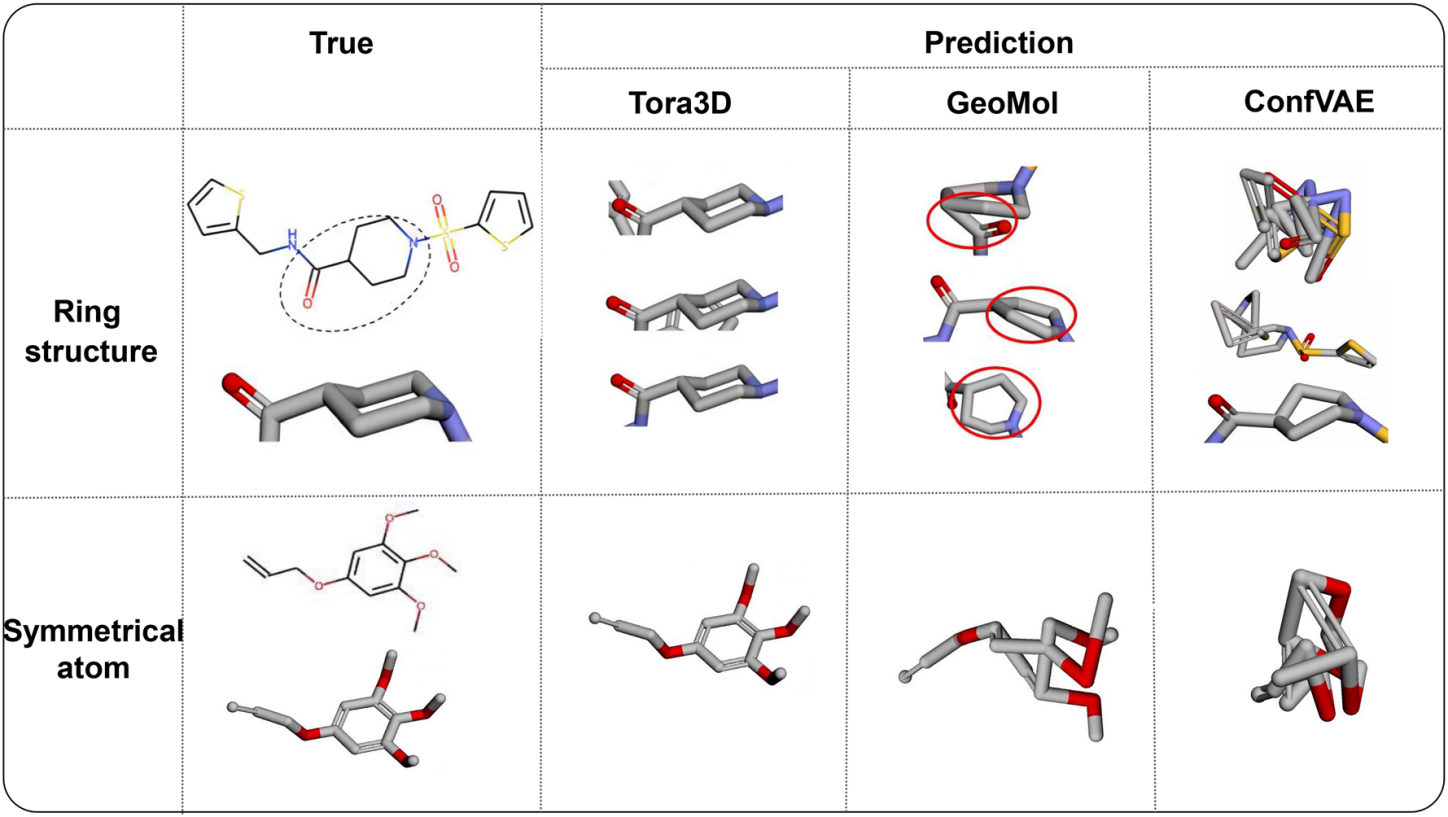
Comparison of conformations predicted by different models. The first column shows the real conformation, and the other columns show the conformations predicted by Tora3D, GeoMol, and ConfVAE, respectively. The first row shows their differences in the predicted structure (hexahydropyridine), and the second row shows their differences in the predicted symmetrical structure (1,2,3-trimethoxybenzene)

Using fixed local structures can avoid the prediction error for symmetric graph nodes (i.e., nodes with the same topology in graph) and ring structures that seriously undermines the accuracy of many 3D prediction model. For example, GeoMol and ConfVAE generate invalid conformation of non-planar rings, such as hexahydropyridine shown in Fig. 6. GeoMol explicitly models and predicts bond angles and length, but the accumulated errors cause flattened or severely distorted ring; the distance matrix used in ConfVAE is difficult to enforce geometric graph constraint and inevitably lead to seriously implausible structures. Whereas Tora3D could correctly maintain the chair conformation that conforms to the chemical rules by assembling from the initial conformation. Another case is predicting pairs of atoms that are completely structurally symmetrical in a molecule. The classical message-passing neural networks (MPNNs) will embed symmetric graph nodes to the same point in the embedding space and thus generate identical coordinates for them. Previous works often add noise, augment atom features or design complex loss functions to avoid the overlapping of symmetric graph nodes [30]. In the case of the 1,2,3-trimethoxybenzene group (Fig. 6), though appending initial random noise feature vectors does avoid the overlapping of the symmetrical benzene ring and the methoxyl groups in GeoMol and ConfVAE, as Ganea et al. stated that symmetric graph nodes that are less than 3 hops away are indistinguishable by MPNNs in general, the matching information between the ring plane of benzene with the ground true conformation nodes is lost.

Hence, as the accuracy of local structures significantly impacts many models’ performance, Tora3D reconstructing the conformation by a two-stage generation procedure that utilizes predicted torsion angles to assemble fixed local structures can be of practical value. Even if a conformation generated by Tora3D is not in the provided conformation set, it still conforms to the chemical rules and is thus valid and usable.

### Energy-guided conformational generation

**Fig. 7:**
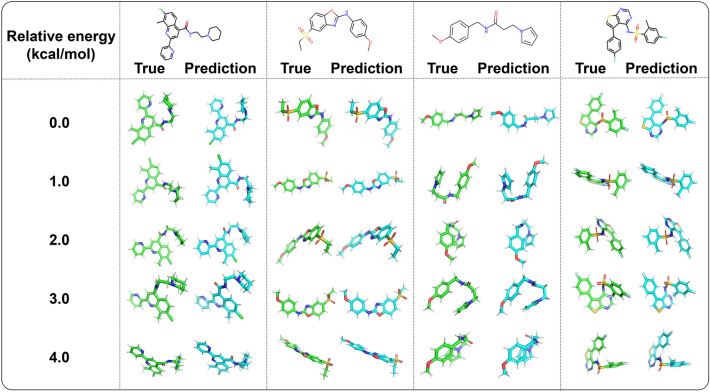
The predicted results of Tora3D. The relative energy is the absolute energy of a conformation minus the absolute energy of the lowest-energy conformation. And 0 kcal/mol indicates the lowest-energy conformation. The true conformations (green) are on the left and the predicted conformations (blue) are on the right of each column (image source: Pymol [32])

Common methods of conformational sampling in machine learning-based models are random initialization and RDKit initialization. An RDKit initialization can achieve better accuracy by providing a more accurate starting point, while a random initialization can achieve better coverage by the sufficient sampling of the space [31]. To promote diverse conformation ensembles with both good coverage and accuracy, a mixture of random initialization and energy-specific input is used in Tora3D for the conformational generation process. In addition to Gaussian noise that is added to T^0^ that allows the model generates multiple conformations, Tora3D can generate a set of conformations with geometrical diversity by varying relative energies as model input. As shown in Fig. 7, the Tora3D predictions of conformations with various relative energies could reproduce the ground true conformations of depicted molecules. The high structural quality, as well as the competitive COV score achieved by Tora3D, suggest that relative energies can be used to guide the generation of a diverse collection of conformations.

## Conclusion

Due to the extension of the application scope of molecular 3D structure in the field of drug development, the methodology of molecular conformation generation continues to develop. Here, combining systematic search methods and advanced deep learning models, we propose a deep learning-based model to predict the torsion angles of rotatable bonds in a molecule, thereby predicting molecular conformations. Tora3D is superior to a series of baseline models with comparatively high accuracy but does not sacrifice efficiency. In the aspect of conformational validity, Tora3D employs an autoregressive approach to predict all torsion angles, so that the problem of the collision between local structures can also be solved in an interpretable way. The autoregressive algorithm could consider every rotatable bond sequentially to avoid clashes among local structures, and further improve the spatial rationality of the whole molecule by attention mechanism. At the same time, reconstructing the conformation by a two-stage generation procedure avoids many invalid local structures. In the aspect of conformational diversity, by varying relative energies as model input, Tora3D can generate energy-specific conformation ensemble with good coverage. In addition, as an improvement in model structure to promote accuracy, we proposed a new method of position encoding on graphs that compensates for the difficulties of traditional GNN long-distance messaging. The ablation test of the position vector verified that Tora3D outperformed traditional GNN to solve the problem of long-distance information passing.

Tora3D is a promising tool to generate valid and diverse molecular conformation sets with competitive accuracy and efficiency. Its performance is particularly high for drug-like molecules with rotatable bonds less than 10. Especially, energy-guided conformational generation provides many possibilities for model application in the field of drug design, as conformational energy is crucial to understand how a molecule binds to a specific target protein. In future work, we will do more rigorous explorations and we expect that Tora3D will be applied in a variety of downstream tasks including large-scale virtual screening, molecular property prediction, and drug-target interaction prediction, thus speeding up areas of drug discovery.

## Supplementary Information

**Additional file 1**: **Figure S1.** The distribution of the number of conformations.**Figure S2.** The true and predicted conformations for chiralmolecule, spirans and macrocycles. **Figure S3.** The comparison of thetorsion angles predicted by Tora3D with the corresponding statisticaldistribution provided by Torsion Library. **Figure S4.** The conformationcomparison between Tora3D and Conformator. **Table S1.** Hyperparameters.**Table S2.** The prediction performance for chiral molecules, spiransand macrocycles. **Table S3.** Performance comparison with TorsionNET onthe GEOM-drugs dataset (Test set II). **Table S4.** Performance comparisonwith Conformator’s initial conformations on the GEOM-drugs dataset (Testset II). **Implementation of Torsion Library. Implementation of TorsionNet.The comparison between the initial conformations and Tora3D’s generatedconformations. Loss** Tora3D’s Loss.

## Data Availability

The supplementary materials provide the distribution of data sets, some hyperparameters of the model, the settings of loss functions and the implementation and results of some model comparisons. The source code and related datasets are provided for academic use: https://github.com/zimeizhng/Tora3D.

## Abbreviations

3D: Three-dimensional
DFT: Density functional theory
QSAR: Quantitative structure–activity relationships
MC: Monte Carlo simulation
DG: Distance geometry
GA: Genetic Algorithm
WL: Weisfeiler-Lehman
GNN: Graph neural network
MPNN: Message-passing neural network
nRotb: Number of rotatable bonds
