# Supplementary material for: Tora3D: an autoregressive torsion angle prediction model for molecular 3D conformation generation
Source: J Cheminform. 2023 Jun 7;15:57. doi: 10.1186/s13321-023-00726-8 (PMC10245430; doi:10.1186/s13321-023-00726-8)
Supplement: Supplementary file 1 — Additional file 1: Figure S1. The distribution of the number of conformations.Figure S2. The true and predicted conformations for chiralmolecule, spirans and macrocycles. Figure S3. The comparison of thetorsion angles predicted by Tora3D with the corresponding statisticaldistribution provided by Torsion Library. Figure S4. The conformationcomparison between Tora3D and Conformator. Table S1. Hyperparameters.Table S2. The prediction performance for chiral molecules, spiransand macrocycles. Table S3. Performance comparison with TorsionNET onthe GEOM-drugs dataset (Test set II). Table S4. Performance comparisonwith Conformator’s initial conformations on the GEOM-drugs dataset (Testset II). Implementation of Torsion Library. Implementation of TorsionNet.The comparison between the initial conformations and Tora3D’s generatedconformations. Loss Tora3D’s Loss. [file 13321_2023_726_MOESM1_ESM.docx]

Supporting Information

Tora3D: An autoregressive torsion angle prediction model for molecular 3D conformation generation

Zimei Zhang[1,2], Gang Wang[2,3], Rui Li[5,2], Lin Ni[4,2], RunZe Zhang[2,3], Kaiyang Cheng[4,2], Qun Ren[4,2], Xiangtai Kong[2,3], Shengkun Ni[2,3], Xiaochu Tong[2,3], Li Luo[7], Dingyan Wang[6], Xiaojie Lu[2,3], Mingyue Zheng[1,2,3,4*], and Xutong Li[2,3*]

[1] Division of Life Science and Medicine, University of Science and Technology of China, Hefei 230026, Anhui, China

[2] Drug Discovery and Design Center, State Key Laboratory of Drug Research, Shanghai Institute of Materia Medica, Chinese Academy of Sciences, 555 Zuchongzhi Road, Shanghai 201203, China

[3] University of Chinese Academy of Sciences, No.19A Yuquan Road, Beijing 100049, China

[4] Nanjing University of Chinese Medicine, 138 Xianlin Road, Nanjing 210023, China

[5] School of Pharmacy, China Pharmaceutical University, 639 Longmian Road, Nanjing 211198, China

[6] Lingang Laboratory, Shanghai 200031, China

[7] Precision Pharmacy & Drug Development Center, Department of Pharmacy, Tangdu Hospital, Fourth Military Medical University, Xi'an 710038, China

[*] Correspondence: myzheng@simm.ac.cn; lixutong@simm.ac.cn

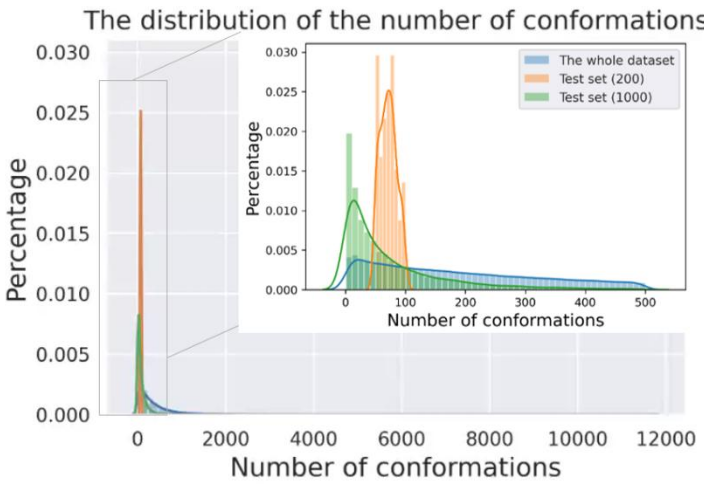

Figure S1. The distribution of the number of conformations. (a) The orange curve is the distribution of the number of conformations in the test set of 200 molecules used in the prior works. The green curve is the distribution of the number of conformations in our large-scale dataset. The blue curve is the real distribution of the conformation numbers of the GEOM-drugs.

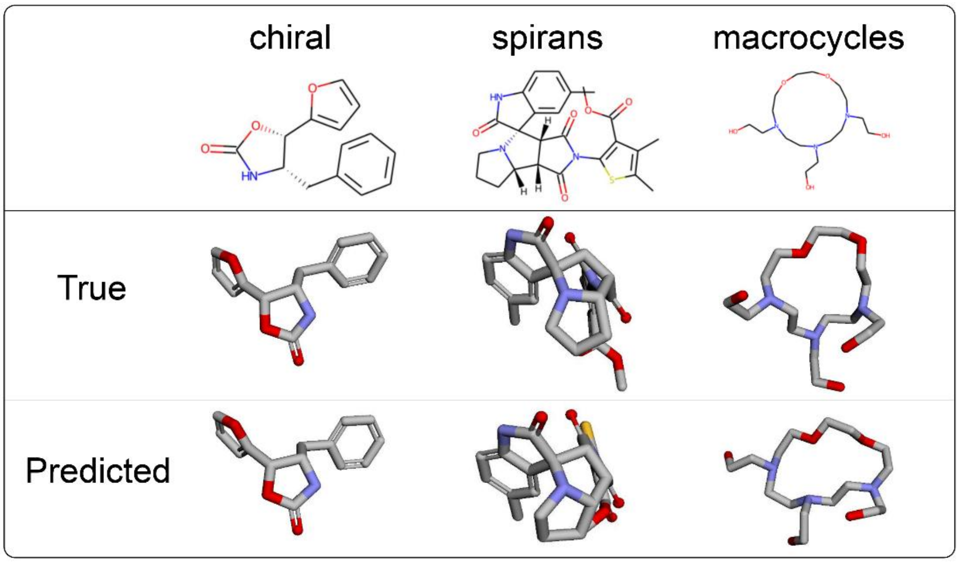

Figure S2. The true and predicted conformations for chiral molecule, spirans and macrocycles.

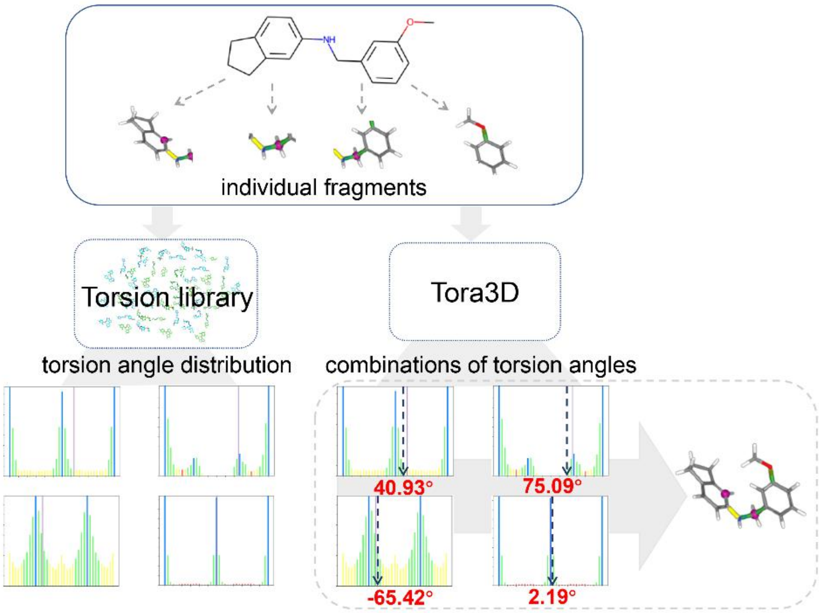

Figure S3. The comparison of the torsion angles predicted by Tora3D with the corresponding statistical distribution provided by Torsion Library.

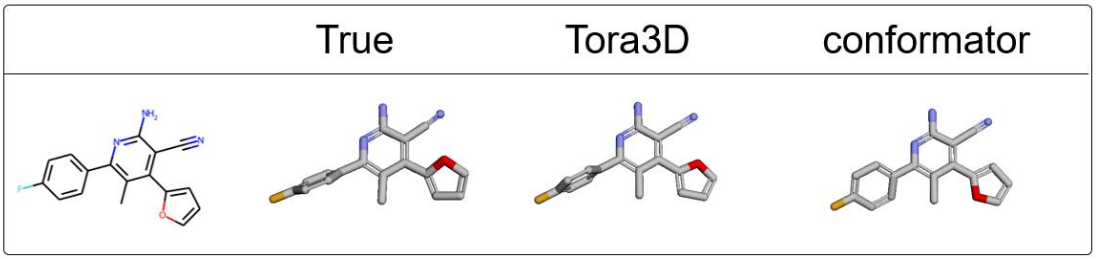

Figure S4. The conformation comparison between Tora3D and Conformator.

Table S1. Hyperparameters

Symbol	value
The mean of Gaussian noise	0
The standard deviation of Gaussian noise	5
T	1
N	1
M	3

Table S2. The prediction performance for chiral molecules, spirans and macrocycles

	COV-R(↑)	MAT-R(↓)	COV-P(↑)	MAT-P(↓)
chiral molecules	57.46	1.2057	43.84	1.5093

spirans	88.43	0.8070	82.85	0.9140
macrocycles	15.65	1.4249	70.46	1.1776

Table S3. Performance comparison with TorsionNet on the GEOM-drugs dataset (Test set II).

models	COV-R(↑)	MAT-R(↓)	COV-P(↑)	MAT-P(↓)	Speed (s/molecule)
TorsionNet	80.03	0.9483	**66.76**	**1.0855**	830+
Tora3D	**80.37**	**0.9272**	62.22	1.1524	**5 - 8.4**

Table S4. Performance comparison with Conformator' s initial conformations on the GEOM-drugs dataset (Test set II).

models	Average minimum RMSD(↓)
Conformator's initial conformations	0.9217
Tora3D	**0.4607**

Implementation of Torsion Library

We have compared the torsion angles predicted by Tora3D with the corresponding statistical distribution provided by Torsion Library with a case. Specifically, first, a molecule was cut into several fragments. Then, the torsion angle distribution of these fragments can be found in Torsion library. At the same time, Tora3D predicts the torsion angles of them. At last, the torsion angles predicted by Tora3D were examined for their position in the distribution. As shown in Figure S3, torsion angles are affected by the molecular environment and may not necessarily be the energy-minimized angle (the peak), but rationally lie in the range of their corresponding distribution.

Implementation of TorsionNet

As TorsionNet can be used to prioritize low-energy conformations, we generated an ensemble of random conformations of the molecules in Test set II and use TorsionNet to rank them. Specifically, first, 1000 random conformations were generated for a molecule, by setting each rotatable bond of an initial conformation to 36 equally spaced points (separated by 10°) over the entire −180° to +180° range (i.e. [-180°, …, -20°, -10°, 0°, 10°, 20°, …, 180°]). Then, TorsionNet was used to rank these random conformations to select the top 2k low-energy conformations (k represents the number of ground true conformations). Finally, these low-energy conformations were compared with the ground truth conformations to calculate scoring metrics, which were showed in Figure S3.

Our model shows superior in both the COV-R and MAT-R metrics compared with TorsionNet, but lower COV-P and MAT-P. The Recall metrics concentrate more on the diversity, and the Precision metrics depend more on the quality. The higher COV-R and MAT-R of Tora3D is reasonable as it adds Gaussian noise to initial torsion angle representations that allows the model generates multiple conformations, and varies relative energies in model input to further enhance geometrical diversity of the predicted conformations. TorsionNet selects the top low-energy conformations, which leads to the loss of diversity but the improvement of precision. In addition, our model offered an efficient balance between speed and accuracy, which was much faster than TorsionNet. TorsionNet takes about 14 minutes to obtain one conformation, while our model can complete it in a few seconds. More importantly, as a deep learning model, Tora3D can accurately predict torsion angles to reconstruct an entire conformation, making it possible to generate 3D representation of molecules for downstream tasks such as predicting molecular properties and screening target drugs.

The comparison between the initial conformations and Tora3D ' s generated conformations

For a molecule, we obtained an initial conformation through Conformator, and calculated its RMSDs with reference conformations. The minimum RMSD donates the

different between the initial conformation and the most similar reference conformation with it, which is retained to calculate the average performance of Conformator's initial conformations for all molecules in Test set II. For parallel comparison, the minimum RMSD is calculated between the most similar pairs of the generated conformations and the reference conformation set.

The average minimum RMSD is shown in Table S4, and an initial conformation and its corresponding generated conformation predicted by Tora3D is taken as an example shown in Figure S4. The much lower average minimum RMSD Tora3D's conformations than that of initial conformations indicates that the Tora3D can twist the initial conformation to make it closer to the ground truth conformations. Even the local structures are valid, the accurate prediction of torsion angles through Tora3D is necessary to ensure the rational spatial structure for the molecule as it shown in Table S4.

Loss

The loss function employed in this study is based on comparing the ground truth and predicted torsion angle values of the molecular structure. However, instead of directly using the torsion angle value to calculate the loss, we divide it into two separate targets. This division is necessary because calculating the Mean Squared Error (MSE) directly between the true and predicted torsion angle values can lead to issues. Specifically, when dealing with spatially close angles like -179° and 179° that are only 2 degrees apart, a direct calculation could yield a large loss value, making the model difficult to train effectively. By introducing an additional task of predicting signs, we aim to simplify the training process of the neural network. Consequently, the total loss consists of two components: the MSE loss between the true and predicted absolute values of the torsion angle, and the cross-entropy loss (CE) between the true and predicted sign (either positive or negative) of the torsion angle. We use VALUE to denote the absolute values of the torsion angles, and SIG to denote the positive or negative signs of torsion angles. A molecule with z rotatable bonds has two targets, i.e., $VALUE = \{value_1, value_2, ..., value_z\}$ and $SIG = \{sig_1, sig_2, ..., sig_z\}$, and thus the loss function is

$$LOSS = LOSS_{sig} + LOSS_{value} \tag{1}$$

where $LOSS_{sig}$ uses CE and $LOSS_{value}$ uses MSE.

$$LOSS = CE\left(SIG, \widehat{SIG}\right) + MSE(VALUE, \widehat{VALUE}) \quad (2)$$
